# The chromosomal genome sequence of the sponge, *Rhabdastrella globostellata* (Carter, 1883) (Tetractinellida: Ancorinidae) and its associated microbial metagenome sequences

**DOI:** 10.12688/wellcomeopenres.27093.1

**Published:** 2026-07-21

**Authors:** Sandie M Degnan, Z. Sheen Wong, Bernard M Degnan, Dirk Erpenbeck, Ute Hentschel, Graeme Oatley, Elizabeth Sinclair, Eerik Aunin, Noah Gettle, Camilla Santos, Michael Paulini, Haoyu Niu, Victoria McKenna, Rebecca O’Brien, Wellcome Sanger Institute Tree of Life Management, Samples and Laboratory team, Wellcome Sanger Institute Scientific Operations: Sequencing Operations, Wellcome Sanger Institute Tree of Life Core Informatics team, EBI Aquatic Symbiosis Genomics Data Portal Team, Aquatic Symbiosis Genomics Project Leadership

**Affiliations:** 1 The University of Queensland, Saint Lucia, Queensland, Australia; 2 Department of Earth and Environmental Sciences, Palaeontology & Geobiology,, LMU München, München, Germany; 3 GEOMAR Helmholtz Centre for Ocean Research Kiel, Kiel, Germany; 4 Tree of Life Programme, Wellcome Sanger Institute, Hinxton, England, UK

**Keywords:** Rhabdastrella globostellata, sponge, genome sequence, chromosomal, Tetractinellida, microbial metagenome assembly

## Abstract

We present a genome assembly from an individual *Rhabdastrella globostellata* (sponge; Porifera; Demospongiae; Tetractinellida; Ancorinidae). The genome sequence has a total length of 155.58 megabases. Most of the assembly (98.97%) is scaffolded into 21 chromosomal pseudomolecules. The mitochondrial genome has also been assembled, with a length of 18.12 kilobases. Gene annotation of this assembly by Ensembl identified 17 532 protein-coding genes. From the metagenome data, we recovered 131 bins, of which 55 were high-quality MAGs, representing 19 phyla (i.e., Pseudomonadota, Chloroflexota, Acidobacteriota) and candidate phyla (including *Ca.* Poribacteria).

## Species taxonomy

Eukaryota; Opisthokonta; Metazoa; Porifera; Demospongiae; Heteroscleromorpha; Tetractinellida; Astrophorina; Ancorinidae; *Rhabdastrella*; *Rhabdastrella globostellata* ( Carter, 1883) (NCBI:txid473528).

## Background

The demosponge *Rhabdastrella globostellata* ( Carter, 1883) is widely distributed throughout coastal regions of the tropical and subtropical Indian and Pacific Oceans, including coral reefs and rocky outcrops. It is commonly known as the yellow pot sponge in reference to its generally globular shape up to 45 cm in diameter with apical surface depressions 0.5–5 cm deep, and its colouration that comprises a brown outer layer and a mustard yellow mesohyl ( Carter, 1883). It has attracted extensive interest as a source of numerous bioactive compounds, particularly a variety of cytotoxic isomalabaricanes triterpene molecules that have selective anticancer or antibacterial activity ( Chen *et al.*, 2022; Fouad *et al.*, 2006; Hirashima *et al.*, 2010; Kiem *et al.*, 2018; Kozhushnaya *et al.*, 2023; Li *et al.*, 2010; Tasdemir *et al.*, 2002; Trang *et al.*, 2022).

*R. globostellata* is a high-microbial-abundance (HMA) sponge with a microbiome that includes the HMA-indicator phyla *Chloroflexi* and *Poribacteria* ( Lafi *et al.*, 2009; Maggioni *et al.*, 2023b; Moitinho-Silva *et al.*, 2017; Steinert *et al.*, 2016). Field experiments have demonstrated the capacity of the *R. globostellata* holobiont to uptake both bacterial cells and dissolved organic matter from the surrounding seawater, and to release inorganic nitrogen, at rates similar to those reported in other sponge species ( Hildebrand *et al.*, 2022). This, combined with its high documented abundance at some sites, means that it may play a significant role in ecosystem engineering ( Maggioni *et al.*, 2023a). On the Great Barrier Reef, *R. globostellata* shares its shallow reef flat habitat with the well-characterised LMA sponge *Amphimedon queenslandica.* As such, the genome assemblies provide an excellent opportunity to compare and contrast the molecular mechanisms by which each holobiont deals with a common suite of ecological challenges.

## Methods

### Sample acquisition

A specimen of *Rhabdastrella globostellata* (specimen ID GHC0000198, ToLID odRhaGlob2; Figure 1), was collected at a depth of 1 m on the intertidal reef flat of Heron Reef on the southern Great Barrier Reef (−18.8225667, 147.6375500) on the 2021-01-24 by snorkel. The specimen was transported live in native seawater to the University of Queensland Goddard Building closed aquarium system, where tissue was sampled within 24 hours of removal from the reef, and immediately snap frozen in liquid nitrogen and stored at −80 °C until processing for genome sequencing. The odRhaGlob2 specimen was also used for RNA sequencing.

**Figure 1.**
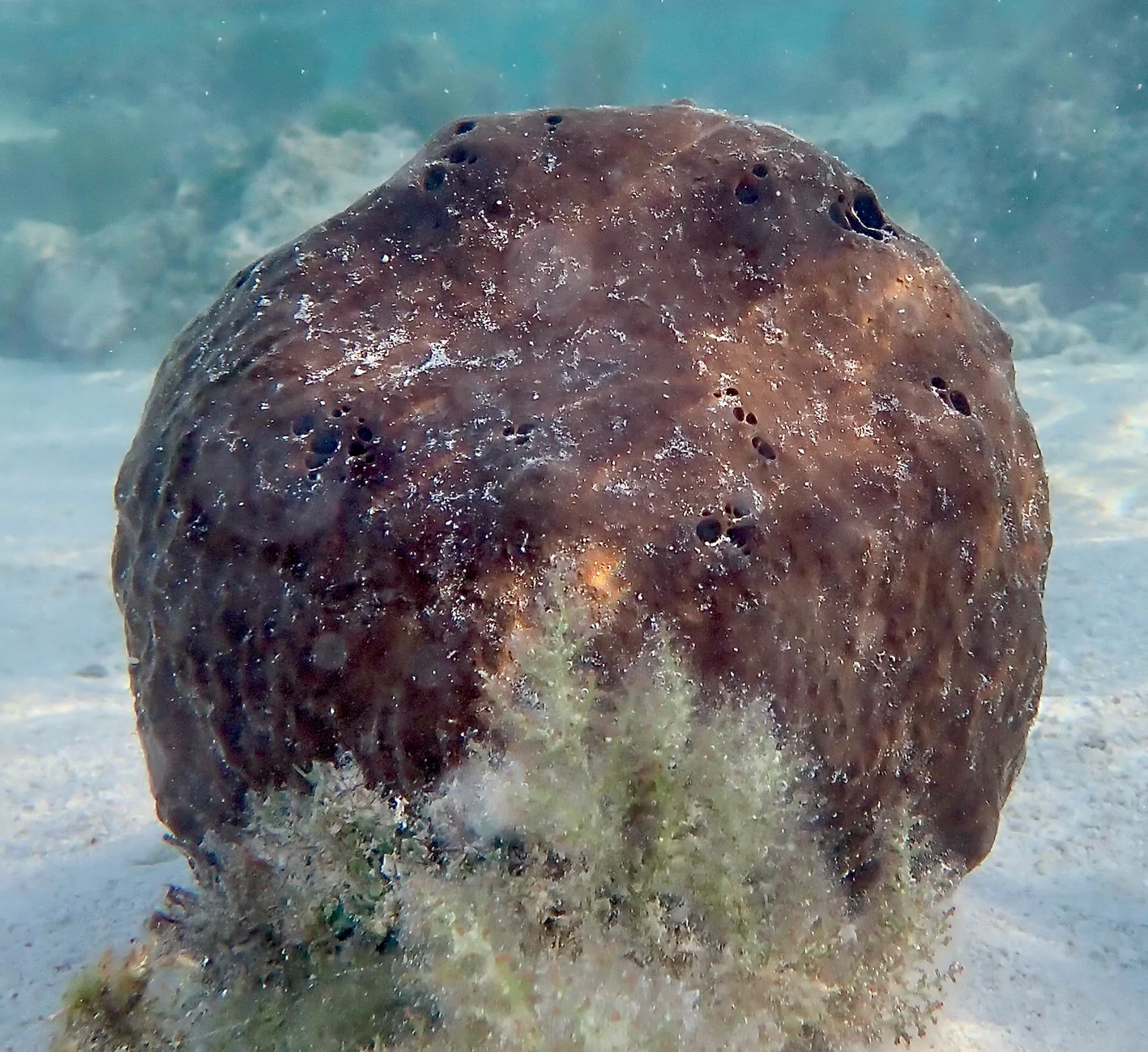
*In-situ* image of *Rhabdastrella globostellata* collected from Heron Reef on the southern Great Barrier Reef.

### Nucleic acid extraction

Protocols for high molecular weight (HMW) DNA extraction developed at the Wellcome Sanger Institute (WSI) Tree of Life Core Laboratory are available on protocols.io ( Howard *et al.*, 2025). The odRhaGlob2 sample was weighed and triaged to determine the appropriate extraction protocol. Tissue was disrupted using the sponge squeezing protocol. HMW DNA was extracted using the Manual MagAttract protocol. DNA was sheared into an average fragment size of 12–20 kb following the Megaruptor®3 for LI PacBio protocol. Sheared DNA was purified by manual SPRI (solid-phase reversible immobilisation). The concentration of the sheared and purified DNA was assessed using a Nanodrop spectrophotometer and Qubit Fluorometer using the Qubit dsDNA High Sensitivity Assay kit. Fragment size distribution was evaluated by running the sample on the FemtoPulse system. For this sample, the final post-shearing DNA had a Qubit concentration of 6.08 ng/μL and a yield of 760.00 ng.

RNA was extracted from tissue of the odRhaGlob2 specimen in the Tree of Life Laboratory at the WSI using the RNA Extraction: Automated MagMax™ *mir*Vana protocol. The RNA concentration was assessed using a Nanodrop spectrophotometer and a Qubit Fluorometer using the Qubit RNA Broad-Range Assay kit. Analysis of the integrity of the RNA was done using the Agilent RNA 6000 Pico Kit and Eukaryotic Total RNA assay.

### PacBio HiFi library preparation and sequencing

Library preparation and sequencing were performed at the WSI Scientific Operations core. Libraries were prepared using the SMRTbell Prep Kit 3.0 (Pacific Biosciences, California, USA), following the manufacturer’s instructions. The kit includes reagents for end repair/A-tailing, adapter ligation, post-ligation SMRTbell bead clean-up, and nuclease treatment. Size selection and clean-up were performed using diluted AMPure PB beads (Pacific Biosciences). DNA concentration was quantified using a Qubit Fluorometer v4.0 (ThermoFisher Scientific) and the Qubit 1X dsDNA HS assay kit. Final library fragment size was assessed with the Agilent Femto Pulse Automated Pulsed Field CE Instrument (Agilent Technologies) using the gDNA 55 kb BAC analysis kit.

The sample was sequenced on a Revio instrument (Pacific Biosciences, California, USA). Prepared libraries with a molarity greater than 2 nM were normalised to 2 nM, and 15 μL was used for making complexes. For libraries with a molarity below 2 nM, the available 10 μL library volume was used without normalisation. Primers were annealed and polymerases were bound to generate circularised complexes, following the manufacturer’s instructions. Complexes were purified using 1.2× SMRTbell beads, diluted to the Revio loading concentration of 200–300 pM, and spiked with a Revio sequencing internal control. The sample was sequenced on a Revio 25 M SMRT cell. SMRT Link software (Pacific Biosciences), a web-based workflow manager, was used to configure and monitor the run and to carry out primary and secondary data analysis.

### Hi-C

***Sample preparation and crosslinking***

The Hi-C sample was prepared from 20–50 mg of frozen tissue from the odRhaGlob2 sample using the Arima-HiC v2 kit (Arima Genomics). Following the manufacturer’s instructions, tissue was fixed and DNA crosslinked using TC buffer to a final formaldehyde concentration of 2%. The tissue was homogenised using the Diagnocine Power Masher-II. Crosslinked DNA was digested with a restriction enzyme master mix, biotinylated, and ligated. Clean-up was performed with SPRISelect beads before library preparation. DNA concentration was measured with the Qubit Fluorometer (Thermo Fisher Scientific) and Qubit HS Assay Kit. The biotinylation percentage was estimated using the Arima-HiC v2 QC beads.

***Hi-C library preparation and sequencing***

Biotinylated DNA constructs were fragmented using a Covaris E220 sonicator and size selected to 400–600 bp using SPRISelect beads. DNA was enriched with Arima-HiC v2 kit Enrichment beads. End repair, A-tailing, and adapter ligation were carried out with the NEBNext Ultra II DNA Library Prep Kit (New England Biolabs), following a modified protocol where library preparation occurs while DNA remains bound to the Enrichment beads. Library amplification was performed using KAPA HiFi HotStart mix and a custom Unique Dual Index (UDI) barcode set (Integrated DNA Technologies). Depending on sample concentration and biotinylation percentage determined at the crosslinking stage, libraries were amplified with 10–16 PCR cycles. Post-PCR clean-up was performed with SPRISelect beads. Libraries were quantified using the AccuClear Ultra High Sensitivity dsDNA Standards Assay Kit (Biotium) and a FLUOstar Omega plate reader (BMG Labtech).

Prior to sequencing, libraries were normalised to 10 ng/μL. Normalised libraries were quantified again to create equimolar and/or weighted 2.8 nM pools. Pool concentrations were checked using the Agilent 4200 TapeStation (Agilent) with High Sensitivity D500 reagents before sequencing. Sequencing was performed using paired-end 150 bp reads on the Illumina NovaSeq 6000.

### RNA library preparation and sequencing

Libraries were prepared using the NEBNext® Ultra II Directional RNA Library Prep Kit for Illumina (New England Biolabs), following the manufacturer’s instructions. Poly(A) mRNA was isolated from 100 ng of total RNA using oligo (dT) beads, converted to cDNA, and uniquely indexed; 14 PCR cycles were performed. Libraries were prepared with an intended fragment size of 100–300 bp. Libraries were quantified, normalised, and pooled equimolarly to a final concentration of 2.8 nM before dilution to 150 pM for loading. Sequencing was carried out on the Illumina NovaSeq 6000, generating paired-end reads.

### Genome assembly

Prior to assembly of the PacBio HiFi reads, a database of *k*-mer counts ( *k* = 31) was generated from the filtered reads using FastK. GenomeScope2 ( Ranallo-Benavidez *et al.*, 2020) was used to analyse the *k*-mer frequency distributions, providing estimates of genome size, heterozygosity, and repeat content.

The HiFi reads were assembled using Hifiasm ( Cheng *et al.*, 2021) with the --primary option. Haplotypic duplications were identified and removed using purge_dups ( Guan *et al.*, 2020). The Hi-C reads ( Rao *et al.*, 2014) were mapped to the primary contigs using bwa-mem2 ( Vasimuddin *et al.*, 2019), and the contigs were scaffolded in YaHS ( Zhou *et al.*, 2023) with the --break option for handling potential misassemblies. The scaffolded assemblies were evaluated using Gfastats ( Formenti *et al.*, 2022), BUSCO ( Manni *et al.*, 2021) and MERQURY.FK ( Rhie *et al.*, 2020).

The mitochondrial genome was assembled using MitoHiFi ( Uliano-Silva *et al.*, 2023).

### Assembly curation

The assembly was decontaminated using the Assembly Screen for Cobionts and Contaminants ( ASCC) pipeline. TreeVal was used to generate the flat files and maps for use in curation. Manual curation was conducted primarily in PretextView and HiGlass ( Kerpedjiev *et al.*, 2018). Scaffolds were visually inspected and corrected as described by Howe *et al*. (2021). Manual corrections included 106 breaks, 106 joins, and removal of 49 haplotypic duplications. This reduced the scaffold count by 75.0%, increased the scaffold N50 by 49.9%, and reduced the total assembly length by 68.4%. The curation process is described at https://gitlab.com/wtsi-grit/rapid-curation . PretextSnapshot was used to generate a Hi-C contact map of the final assembly.

### Assembly quality assessment

The Merqury.FK tool ( Rhie *et al.*, 2020) was run in a Singularity container ( Kurtzer *et al.*, 2017) to evaluate *k*-mer completeness and assembly quality for the primary and alternate haplotypes using the *k*-mer database ( *k* = 31) computed prior to genome assembly. The analysis outputs included assembly QV scores and completeness statistics.

The genome was analysed using the BlobToolKit pipeline, a Nextflow implementation of the earlier Snakemake version ( Challis *et al.*, 2020). The pipeline aligns PacBio reads using minimap2 ( Li, 2018) and SAMtools ( Danecek *et al.*, 2021) to generate coverage tracks. It runs BUSCO ( Manni *et al.*, 2021) using lineages identified from the NCBI Taxonomy ( Schoch *et al.*, 2020). For the three domain-level lineages, BUSCO genes are aligned to the UniProt Reference Proteomes database ( Bateman *et al.*, 2023) using DIAMOND blastp ( Buchfink *et al.*, 2021). The genome is divided into chunks based on the density of BUSCO genes from the closest taxonomic lineage, and each chunk is aligned to the UniProt Reference Proteomes database with DIAMOND blastx. Sequences without hits are chunked using seqtk and aligned to the NT database with blastn ( Altschul *et al.*, 1990). The BlobToolKit suite consolidates all outputs into a blobdir for visualisation. The BlobToolKit pipeline was developed using nf-core tooling ( Ewels *et al.*, 2020) and MultiQC ( Ewels *et al.*, 2016), with containerisation through Docker ( Merkel, 2014) and Singularity ( Kurtzer *et al.*, 2017).

### Metagenome assembly

The metagenome assembly was generated using MetaMDBG ( Benoit *et al.*, 2024). The resulting bin sets of each binning algorithm were optimised and refined using MAGScoT ( Rühlemann *et al.*, 2022). PROKKA ( Seemann, 2014) was used to identify tRNAs and rRNAs in each bin, CheckM ( Parks *et al.*, 2015) (checkM_DB release 2015-01-16) was used to assess bin completeness/contamination, and GTDB-Tk ( Chaumeil *et al.*, 2022) (GTDB release 214) was used to taxonomically classify bins. Taxonomic replicate bins were identified using dRep ( Olm *et al.*, 2017) with default settings (95% ANI threshold). All bins were assessed for quality and categorised as metagenome-assembled genomes (MAGs) if they met the following criteria: contamination ≤5%, presence of 5S, 16S, and 23S rRNA genes, at least 18 unique tRNAs, and either ≥90% completeness or ≥ 50% completeness with fully circularised chromosomes ( Bowers *et al.*, 2017). Bins that did not meet these thresholds, or were identified as taxonomic replicates of MAGs, were retained as ‘binned metagenomes’ provided they had ≥50% completeness and ≤ 10% contamination. A taxonomic tree of the bins was constructed from NCBI classifications using ete3 ( Huerta-Cepas *et al.*, 2016) and visualised with matplotlib.

## Genome sequence report

### Sequence data

PacBio sequencing of the *Rhabdastrella globostellata* specimen generated 189.84 Gb (gigabases) from 23.78 million reads, which were used to assemble the genome. Based on the estimated genome size, the sequencing data provided approximately 180× coverage. Hi-C sequencing produced 196.30 Gb from 650.00 million reads, which were used to scaffold the assembly. RNA sequencing data were also generated and are available in public sequence repositories. Table 1 summarises the specimen and sequencing details.

**Table 1.** Specimen and sequencing data for *Rhabdastrella globostellata* (BioProject PRJEB77594).

Platform	PacBio HiFi	Hi-C	RNA-seq
**ToLID**	odRhaGlob2	odRhaGlob2	odRhaGlob2
**Specimen ID**	GHC0000198	GHC0000198	GHC0000198
**BioSample (source individual)**	SAMEA10157610	SAMEA10157610	SAMEA10157610
**BioSample (tissue)**	SAMEA10157628	SAMEA10157626	SAMEA10157628
**Instrument**	Revio	Illumina NovaSeq 6000	Illumina NovaSeq 6000
**Run accessions**	ERR13382516; ERR14835876; ERR13382518; ERR13382517	ERR13363481; ERR13363483	ERR13363482
**Read count total**	23.78 million reads	650.00 million read pairs	28.36 million read pairs
**Base count total**	189.84 Gb	196.30 Gb	8.56 Gb

### Assembly statistics

The primary haplotype was assembled, and contigs corresponding to an alternate haplotype were also deposited in INSDC databases. The final assembly has a total length of 155.58 Mb in 328 scaffolds, with 606 gaps, and a scaffold N50 of 6.52 Mb ( Table 2).

**Table 2.** Genome assembly data for *Rhabdastrella globostellata.*

Genome assembly	Primary assembly
**Assembly name**	odRhaGlob2.1
**Assembly accession**	GCA_964261875.1
**Alternate haplotype accession**	GCA_964261865.1
**Assembly level**	chromosome
**Span (Mb)**	155.58
**Number of chromosomes**	21
**Number of contigs**	934
**Contig N50**	0.8 Mb
**Number of scaffolds**	328
**Scaffold N50**	6.52 Mb
**Organelles**	Mitochondrial genome: 18.12 kb

Most of the assembly sequence (98.97%) was assigned to 21 chromosomal-level scaffolds. These chromosome-level scaffolds, confirmed by Hi-C data, are named according to size ( Figure 2; Table 3).

**Figure 2.**
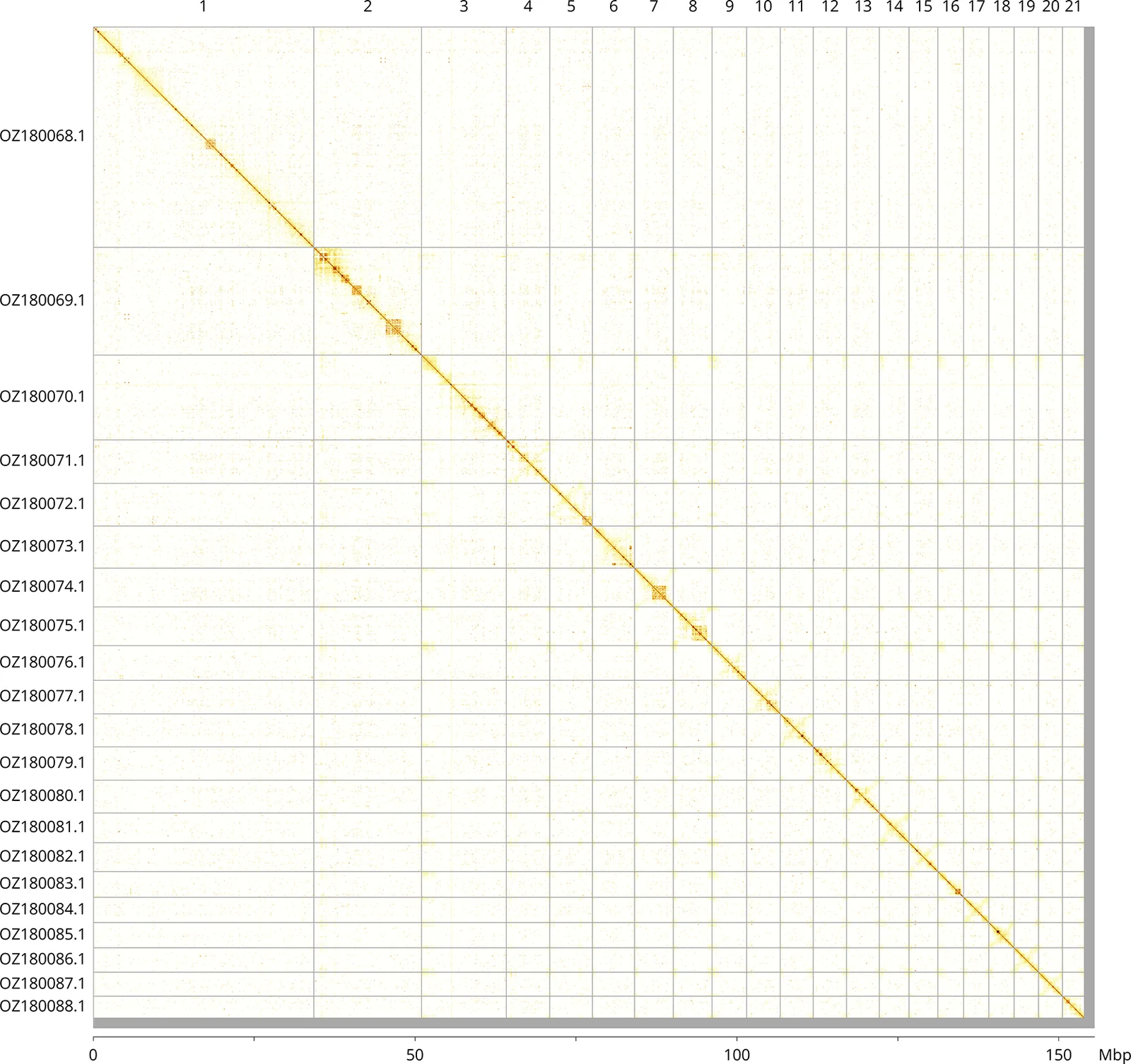
Hi-C contact map of the *Rhabdastrella globostellata* genome assembly. Assembled chromosomes are shown in order of size and labelled along the axes, with a megabase scale shown below. The plot was generated using PretextSnapshot.

**Table 3.** Chromosomal pseudomolecules in the primary genome assembly of *Rhabdastrella globostellata* odRhaGlob2.

INSDC accession	Molecule	Length (Mb)	GC%
OZ180068.1	1	34.30	43
OZ180069.1	2	16.74	43
OZ180070.1	3	13.14	43.50
OZ180071.1	4	6.76	44
OZ180072.1	5	6.65	44.50
OZ180073.1	6	6.52	42.50
OZ180074.1	7	6.04	42.50
OZ180075.1	8	6.04	44.50
OZ180076.1	9	5.36	43
OZ180077.1	10	5.20	42
OZ180078.1	11	5.16	44
OZ180079.1	12	5.14	43
OZ180080.1	13	5.12	42.50
OZ180081.1	14	4.62	44.50
OZ180082.1	15	4.45	43
OZ180083.1	16	4.02	43.50
OZ180084.1	17	3.93	44.50
OZ180085.1	18	3.91	44.50
OZ180086.1	19	3.83	44
OZ180087.1	20	3.69	44
OZ180088.1	21	3.40	43.50

The mitochondrial genome was also assembled (length 18.12 kb, OZ180089.1). This sequence is included as a contig in the multifasta file of the genome submission and as a standalone record.

### Assembly quality metrics

The combined primary and alternate assemblies achieve an estimated Merqury QV of 50.4. BUSCO v.5.5.0 analysis using the metazoa_odb10 reference set ( *n* = 954) identified 60.7% of the expected gene set (single = 60.0%, duplicated = 0.7%). The snail plot in Figure 3 summarises the scaffold length distribution and other assembly statistics for the primary assembly. The blob plot in Figure 4 shows the distribution of scaffolds by GC proportion and coverage.

**Figure 3.**
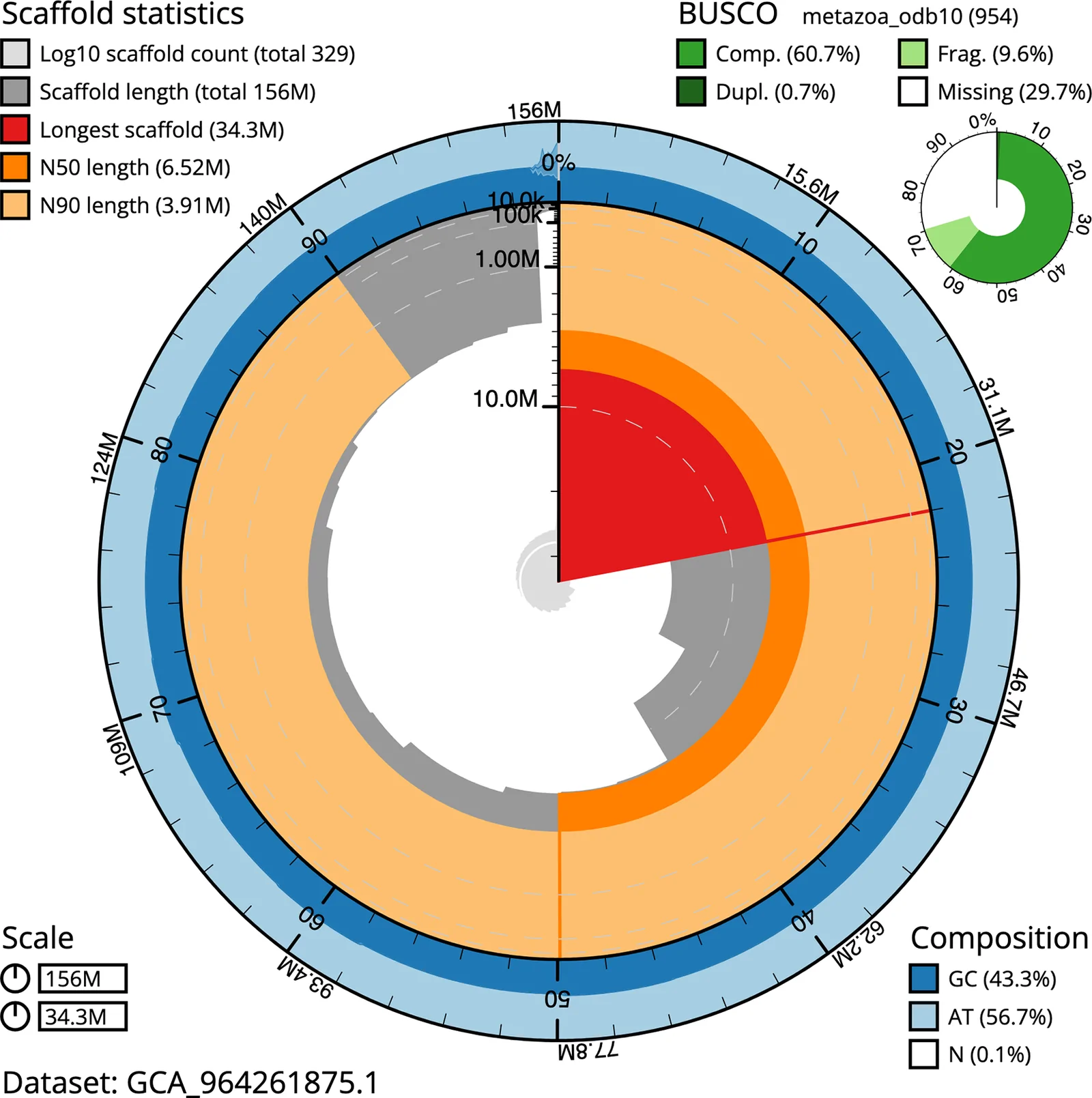
Assembly metrics for odRhaGlob2.1. The BlobToolKit snail plot provides an overview of assembly metrics and BUSCO gene completeness. The circumference represents the length of the whole genome sequence, and the main plot is divided into 1 000 bins around the circumference. The outermost blue tracks display the distribution of GC, AT, and N percentages across the bins. Scaffolds are arranged clockwise from longest to shortest and are depicted in dark grey. The longest scaffold is indicated by the red arc, and the deeper orange and pale orange arcs represent the N50 and N90 lengths. A light grey spiral at the centre shows the cumulative scaffold count on a logarithmic scale. A summary of complete, fragmented, duplicated, and missing BUSCO genes in the metazoa_odb10 set is presented at the top right. An interactive version of this figure can be accessed on the BlobToolKit viewer.

**Figure 4.**
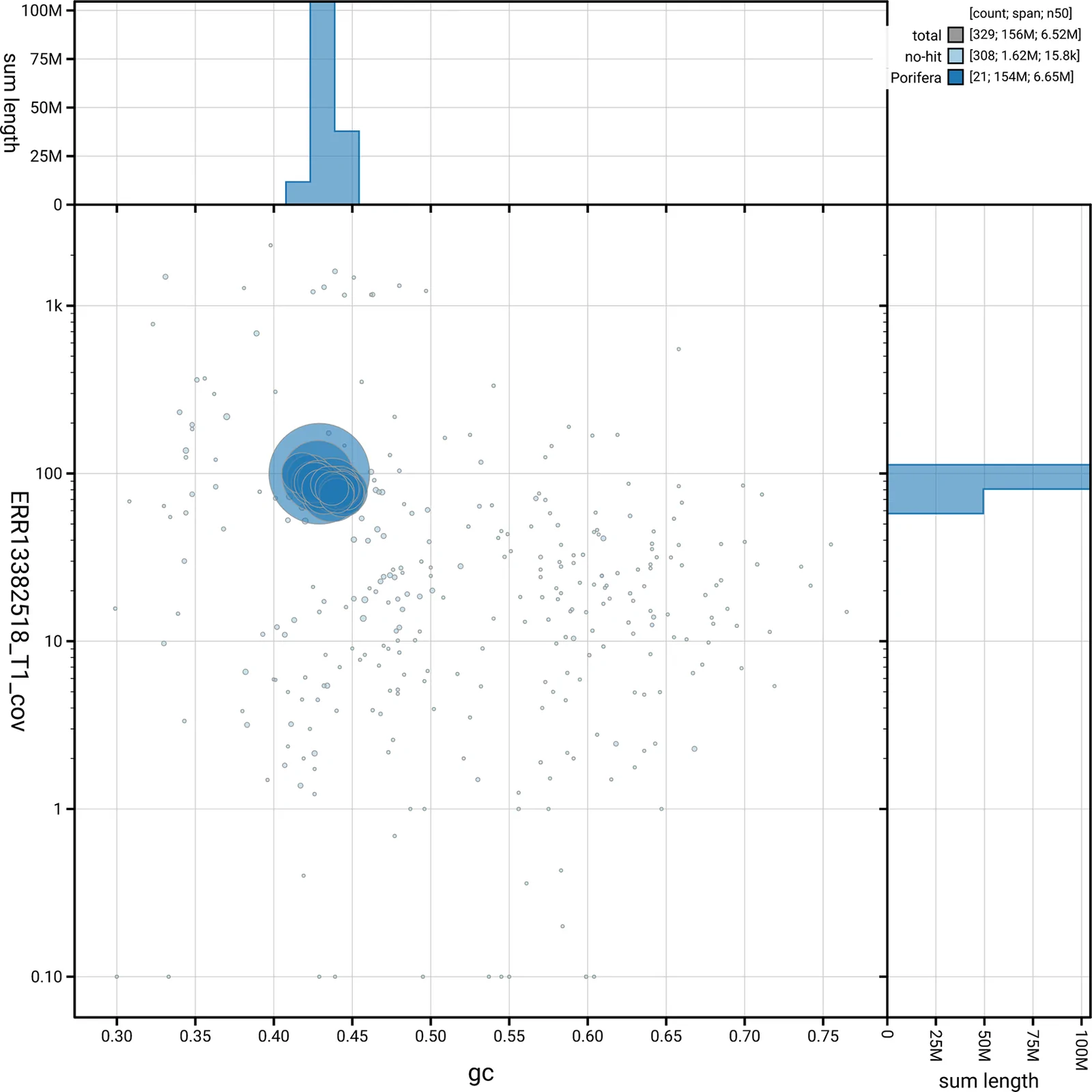
BlobToolKit blob plot for odRhaGlob2.1. The plot shows base coverage (vertical axis) and GC content (horizontal axis). The circles represent scaffolds, with the size proportional to scaffold length and the colour representing phylum membership. The histograms along the axes display the total length of sequences distributed across different levels of coverage and GC content. An interactive version of this figure is available on the BlobToolKit viewer.

Table 4 lists the assembly metric benchmarks adapted from Rhie *et al.* (2021) and the Earth BioGenome Project Report on Assembly Standards January 2026. The EBP metric, calculated for the primary assembly, is **5.C.Q50**.

**Table 4.** Earth Biogenome Project summary metrics for the *Rhabdastrella globostellata* assembly.

Measure	Value	Benchmark
EBP summary (primary)	5.C.Q50	5.C.Q40
Contig N50 length	0.80 Mb	≥ 0.1 Mb
Scaffold N50 length	6.52 Mb	= chromosome N50
Consensus quality (QV)	Primary: 50.0; alternate: 50.5; combined: 50.4	≥ 40
BUSCO	C:60.7% [S:60.0%, D:0.7%], F:9.6%, M:29.7%, n:954	S > 90%; D < 5%
Percentage of assembly assigned to chromosomes	98.97%	≥ 90%

*Note:* The EBP summary uses log10(Contig N50); chromosome-level (C) or log10(Scaffold N50); Q (Merqury QV). BUSCO: C = complete; S = single-copy; D = duplicated; F = fragmented; M = missing; n = orthologues.

**Table 5.** Software versions and sources used for *Rhabdastrella globostellata.*

Software	Version	Source
BLAST	2.14.0	ftp://ftp.ncbi.nlm.nih.gov/blast/executables/blast+/
BlobToolKit	4.3.9	https://github.com/blobtoolkit/blobtoolkit
BUSCO	5.5.0	https://gitlab.com/ezlab/busco
bwa-mem2	2.2.1	https://github.com/bwa-mem2/bwa-mem2
CheckM	2015-01-16	https://ecogenomics.github.io/CheckM/
DIAMOND	2.1.8	https://github.com/bbuchfink/diamond
dRep	3.4.0	https://github.com/MrOlm/drep
ete3	3.1.3	http://etetoolkit.org
fasta_windows	0.2.4	https://github.com/tolkit/fasta_windows
FastK	1.1	https://github.com/thegenemyers/FASTK
GenomeScope2.0	2.0.1	https://github.com/tbenavi1/genomescope2.0
Gfastats	1.3.6	https://github.com/vgl-hub/gfastats
GTDB-Tk	1.2.1	https://github.com/Ecogenomics/GTDBTk
Hifiasm	0.16.1	https://github.com/chhylp123/hifiasm
HiGlass	1.13.4	https://github.com/higlass/higlass
MAGScoT	1.0.0	https://github.com/ikmb/MAGScoT
matplotlib	3.10.3	https://matplotlib.org/
MerquryFK	1.1.0-c1	https://github.com/thegenemyers/MERQURY.FK
MetaMDBG	Pre-release	https://github.com/GaetanBenoitDev/metaMDBG
Minimap2	2.24-r1122	https://github.com/lh3/minimap2
MitoHiFi	2	https://github.com/marcelauliano/MitoHiFi
MultiQC	1.14; 1.17 and 1.18	https://github.com/MultiQC/MultiQC
Nextflow	23.10.0	https://github.com/nextflow-io/nextflow
PretextSnapshot	0.0.5	https://github.com/sanger-tol/PretextSnapshot
PretextView	1.0.3	https://github.com/sanger-tol/PretextView
Prokka	1.14.5	https://github.com/tseemann/prokka
purge_dups	1.2.3	https://github.com/dfguan/purge_dups
samtools	1.19.2	https://github.com/samtools/samtools
sanger-tol/ascc	0.1.0	https://github.com/sanger-tol/ascc
sanger-tol/blobtoolkit	0.6.0	https://github.com/sanger-tol/blobtoolkit
sanger-tol/curationpretext	1.4.2	https://github.com/sanger-tol/curationpretext
Seqtk	1.3	https://github.com/lh3/seqtk
Singularity	3.9.0	https://github.com/sylabs/singularity
TreeVal	1.4.0	https://github.com/sanger-tol/treeval
YaHS	1.1a.2	https://github.com/c-zhou/yahs

### Taxonomic verification

The placement of *Rhabdastrella globostellata* in genus *Rhabdastrella* is currently under discussion ( Cárdenas *et al.*, 2011). We reconstructed a phylogenetic tree with the mitochondrial cytochrome oxidase subunit 1 under all sequences currently published as this species. Unfortunately, holotype data is currently not available for this species. Sequences were aligned using MAFFT v7.450 ( Katoh & Standley, 2013), and analysed with RAxML 8 (GTR + GAMMA+ I model) ( Stamatakis, 2014). The sequence was recovered in a monophyletic *Rhabdastrella globostellata* clade, and identical to verified *R. globostellata* specimens of Cárdenas *et al.* (2011).

### Genome annotation report

The *Rhabdastrella globostellata* genome assembly (GCA_964261875.1) was annotated by Ensembl at the European Bioinformatics Institute (EBI). This annotation includes 29 260 transcribed mRNAs from 17 532 protein-coding and 3 779 non-coding genes. The average transcript length is 4 522.05 bp, with an average of 1.37 coding transcripts per gene and 6.24 exons per transcript. Further details of this annotation are available from the Ensembl annotation page.

### Metagenome report

We recovered 131 bins from the metagenome assembly ( Figure 5), of which 55 met the criteria for MAGs, including 19 fully circularised genomes. The recovered bins represented 19 bacterial and archaeal phyla, with genome sizes ranging from 0.62 to 7.22 Mbp (mean: 3.50 ± 1.41 Mbp). Mean completeness was 82.4% (± 16.0%) with 2.6% (± 2.3%) contamination. Figure 6 summarises the taxa and quality of the metagenome bins. The full per-bin table of taxa and quality metrics for the metagenome bins is available on Zenodo.

**Figure 5.**
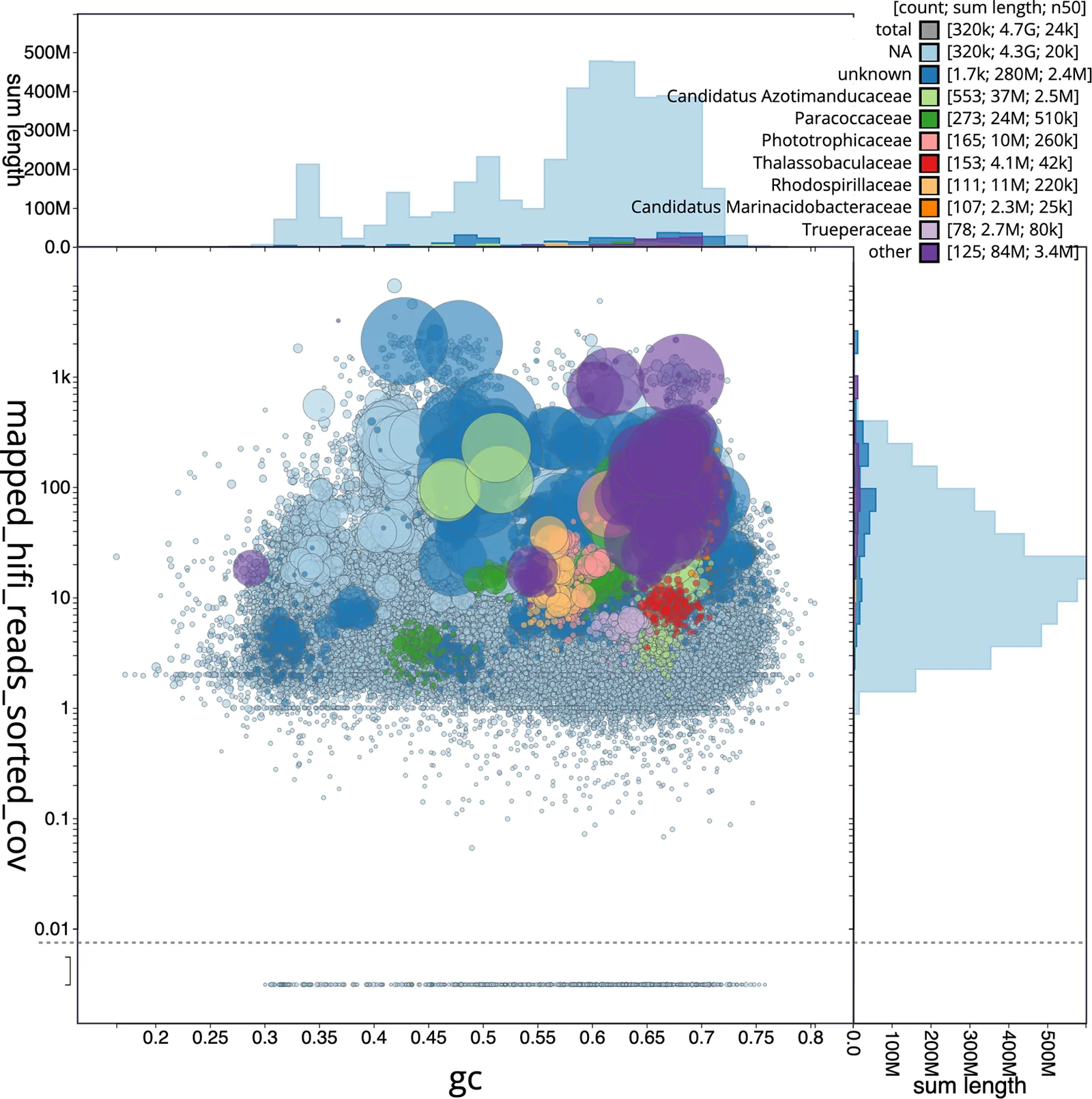
Blob plot for sequences in the *Rhabdastrella globostellata* metagenome. The plot shows base coverage (vertical axis) and GC content (horizontal axis). Binned contigs are coloured by family. Circles are sized in proportion to sequence length on a square-root scale, ranging from 510 to 6 879 066. Histograms show the distribution of sequence length sum along each axis. An interactive version of this figure may be viewed here.

**Figure 6.**
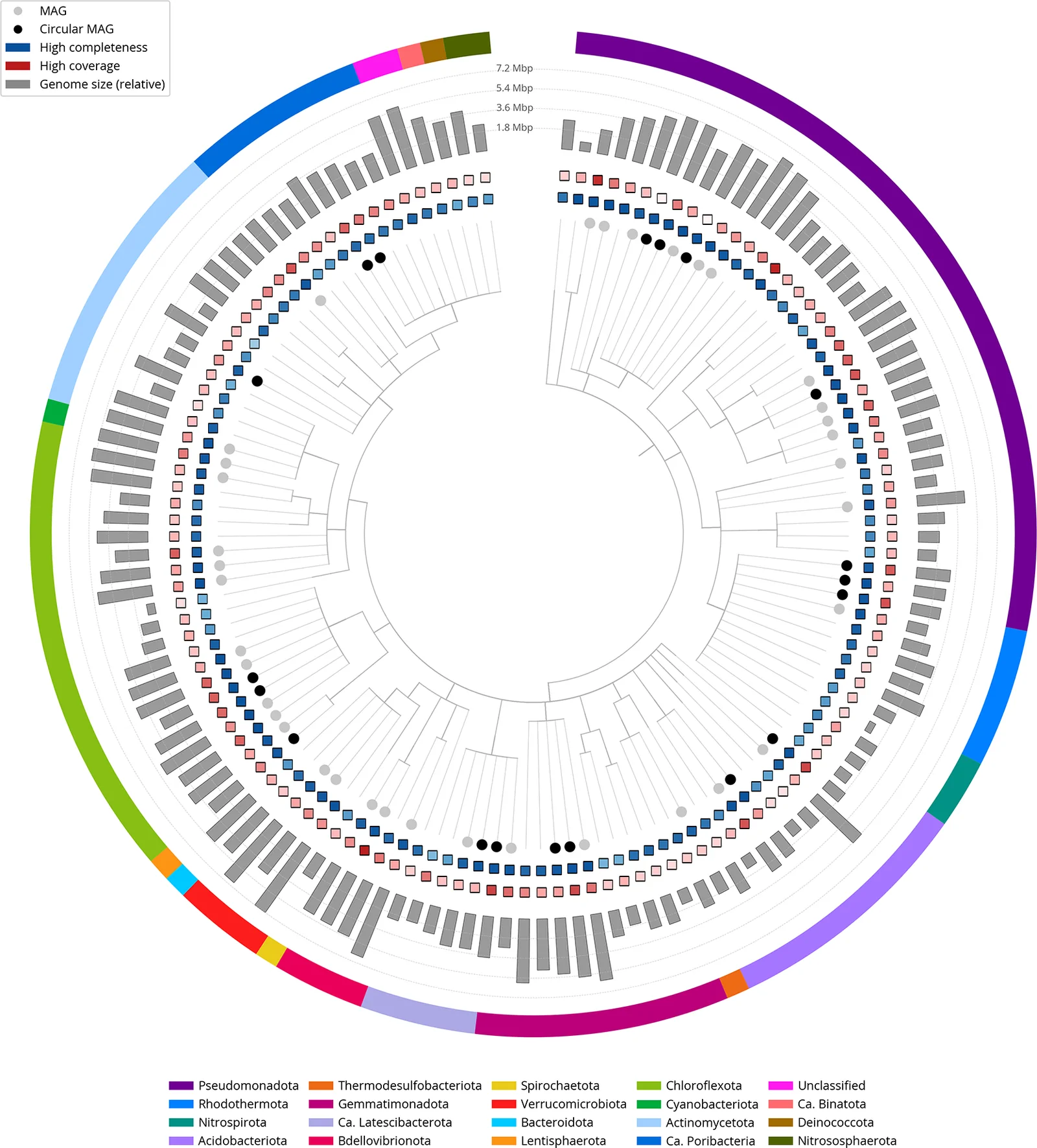
Taxonomic tree based on taxonomic classifications of metagenome bins, constructed using ete3. Colours indicate phylum-level taxonomy. Tracks show genome completeness (blue), sequencing coverage (red, log₁₀), and genome size (grey bars, Mbp). High-quality MAGs are marked with grey circles; fully circularised MAGs in black.

## Author information

Contributors are listed at the following links:

• Members of the Wellcome Sanger Institute Tree of Life Management, Samples and Laboratory team
• Members of Wellcome Sanger Institute Scientific Operations – Sequencing Operations
• Members of the Wellcome Sanger Institute Tree of Life Core Informatics team
• Members of the EBI Aquatic Symbiosis Genomics Data Portal Team
• The Aquatic Symbiosis Genomics Project leadership

## Wellcome Sanger Institute – Legal and Governance

The materials that have contributed to this genome note have been supplied by a Tree of Life collaborator. The Wellcome Sanger Institute employs a process whereby due diligence is carried out proportionate to the nature of the materials themselves, and the circumstances under which they have been/are to be collected and provided for use. The purpose of this is to address and mitigate any potential legal and/or ethical implications of receipt and use of the materials as part of the research project, and to ensure that in doing so we align with best practice wherever possible.

The overarching areas of consideration are:

• Ethical review of provenance and sourcing of the material
• Legality of collection, transfer and use (national and international)

Each transfer of samples is undertaken according to a Research Collaboration Agreement or Material Transfer Agreement entered into by the Tree of Life collaborator, Genome Research Limited (operating as the Wellcome Sanger Institute) and in some circumstances other Tree of Life collaborators.

## Data Availability

European Nucleotide Archive: Rhabdastrella globostellata. Accession number PRJEB77594; https://identifiers.org/ena.embl/PRJEB77594. The genome sequence is released openly for reuse. The *Rhabdastrella globostellata* genome sequencing initiative is part of the Aquatic Symbiosis Genomics Project (PRJEB43743) and the Sanger Institute Tree of Life Programme (PRJEB43745). All raw sequence data and the assembly have been deposited in INSDC databases. Raw data and assembly accession identifiers are reported in Tables 1 and 2. Production code used in genome assembly at the WSI Tree of Life is available at https://github.com/sanger-tol . Table 5 lists software versions used in this study.
