# Supplementary material for: The chromosomal genome sequence of the sponge, Rhabdastrella globostellata (Carter, 1883) (Tetractinellida: Ancorinidae) and its associated microbial metagenome sequences
Source: Wellcome Open Res. 2026 Jul 21;11:459. [Version 1] doi: 10.12688/wellcomeopenres.27093.1 (PMC13675630; doi:10.12688/wellcomeopenres.27093.1)
Supplement: Peer review history

# Reviewer report: Reviewer response for version 1

**DOI:** 10.21956/wellcomeopenres.29823.r167500

**Published:** 2026-10-16

**Authors:** Laura Steindler

**Affiliations:** 1 University of Haifa, Haifa, Haifa, Israel

The manuscript by Degnan and colleagues describes the generation, assembly, and analysis of the nuclear and mitochondrial genomes of the marine sponge *Rhabdastrella globostellata*, together with its associated microbial metagenome. The metagenomic analysis recovered 131 bins, including 55 reported as high-quality MAGs. These genomic resources constitute a valuable addition to the growing number of available sponge genomes and associated microbial metagenomes. The manuscript is clearly presented, and the data appear to be accessible for reuse. Although I am not an expert in eukaryotic genome assembly and the associated bioinformatic procedures, the overall experimental and analytical approaches appear appropriate.

 I identified few points that require clarification or correction:

 1. BUSCO completeness is unusually low (60.7%). The authors should discuss whether this reflects genuine gene loss or substantial sequence divergence in this sponge lineage, an incomplete assembly, or the inadvertent removal of host sequences during decontamination and curation.

 2. The collection coordinates reported in the Methods do not correspond to Heron Reef. Please verify and correct them.

 3. The taxonomic hierarchy places *Rhabdastrella* within Ancorinidae. However, a recently published taxonomic revision (Cárdenas et al., Zoological Journal of the Linnean Society, 2026) supports the transfer of *Rhabdastrella* to Geodiidae and specifically includes *R. globostellata*. Please verify the currently accepted classification and update taxonomic hierarchy accordingly, or explain why Ancorinidae has been retained.

 4. In the Abstract, “i.e.” should be replaced with “e.g.” because the three listed phyla are examples and do not represent all 19 phyla.

Are sufficient details of methods and materials provided to allow replication by others?

Yes

Is the rationale for creating the dataset(s) clearly described?

Yes

Are the datasets clearly presented in a useable and accessible format?

Yes

Are the protocols appropriate and is the work technically sound?

Yes

Reviewer Expertise:

Sponge symbiosis, microbial physiology, microbial comparative genomics, holobiont metatranscriptomics

I confirm that I have read this submission and believe that I have an appropriate level of expertise to confirm that it is of an acceptable scientific standard.

# Reviewer report: Reviewer response for version 1

**DOI:** 10.21956/wellcomeopenres.29823.r165788

**Published:** 2026-09-21

**Authors:** Soo-Je Park

**Affiliations:** 1 Jeju National University, Jeju-si, Jeju-do, South Korea

The manuscript provides a useful genomic resource for *Rhabdastrella globostellata*, including a chromosome-level host genome assembly and associated microbial metagenome-assembled genomes. The overall bioinformatic workflow is clearly described, and software versions are provided in Table 5. However, some additional methodological details would improve the reproducibility of the analyses.

 In particular, I suggest providing the main parameters or command-line options used for the genome and metagenome analyses, especially for tools such as Hifiasm, purge_dups, YaHS, MetaMDBG, MAGScoT, CheckM, GTDB-Tk, and dRep. For software reported as a pre-release version, such as MetaMDBG, a more specific release identifier or commit information would also be helpful. Relevant database versions and key settings used for phylogenetic analyses should likewise be specified where appropriate.

 These details could be provided concisely in the Methods, a supplementary table, or through the publicly available analysis scripts. This would further improve the reproducibility and reuse of this valuable genomic dataset.

Are sufficient details of methods and materials provided to allow replication by others?

Yes

Is the rationale for creating the dataset(s) clearly described?

Yes

Are the datasets clearly presented in a useable and accessible format?

Yes

Are the protocols appropriate and is the work technically sound?

Yes

Reviewer Expertise:

ecogenomics, metagenomics, environmental microbiology

I confirm that I have read this submission and believe that I have an appropriate level of expertise to confirm that it is of an acceptable scientific standard.
